# Retrieving Equivalent Shear Viscosity for Molten Polymers from 3-D Nonisothermal Capillary Flow Simulation

**DOI:** 10.3390/polym13234094

**Published:** 2021-11-24

**Authors:** Yu-Ho Wen, Chen-Chieh Wang, Guo-Sian Cyue, Rong-Hao Kuo, Chia-Hsiang Hsu, Rong-Yeu Chang

**Affiliations:** CoreTech System (Moldex3D) Co., Ltd., Chupei, Hsinchu 302082, Taiwan; ricwen@moldex3d.com (Y.-H.W.); henrycyue@moldex3d.com (G.-S.C.); howardkuo@moldex3d.com (R.-H.K.); davidhsu@moldex3d.com (C.-H.H.); rychang@moldex3d.com (R.-Y.C.)

**Keywords:** shear viscosity, viscous heating, temperature rise, polymer melt, capillary rheometer, nonisothermal simulation, cross model, injection molding

## Abstract

For highly viscous polymer melts, considerable fluid temperature rises produced by viscous heating can be a disturbing factor in viscosity measurements. By scrutinizing the experimental and simulated capillary pressure losses for polymeric liquids, we demonstrate the importance of applying a viscous heating correction to the shear viscosity, so as to correct for large errors introduced by the undesirable temperature rises. Specifically, on the basis of a theoretical derivation and 3-D nonisothermal flow simulation, an approach is developed for retrieving the equivalent shear viscosity in capillary rheometry, and we show that the shear viscosity can be evaluated by using the average fluid temperature at the wall, instead of the bulk temperature, as previously assumed. With the help of a viscous Cross model in analyzing the shear-dominated capillary flow, it is possible to extract the viscous heating contribution to capillary pressure loss, and the general validity of the methodology is assessed using the experiments on a series of thermoplastic melts, including polymers of amorphous, crystalline, and filler-reinforced types. The predictions of the viscous model based on the equivalent viscosity are found to be in good to excellent agreement with experimental pressure drops. For all the materials studied, a near material-independent scaling relation between the dimensionless temperature rise (Θ) and the Nahme number (Na) is found, Θ ~ Na^0.72^, from which the fluid temperature rise due to viscous heating as well as the resultant viscosity change can be predicted.

## 1. Introduction

Shear viscosity is particularly important for thermally developing flow problems in which the temperature, pressure, and velocity in conduits are continually changing in the flow direction. For fast flows of polymeric liquids, the apparent decrease in viscosity may result from shear thinning (decrease in viscosity because of non-Newtonian effects) [1] as well as from temperature thinning (decrease in viscosity because of temperature rise caused by viscous heating) [2,3,4]. In most flow problems, viscous heating is not important. However, in injection molding of thin-walled parts, wall shear rates can significantly exceed 1000 $s^{-1}$ and intense viscous heating arises, thus resulting in a severely reduced viscosity [5]. For a simulation of such injection molded parts, an excellent description of the rheological behavior of viscous polymer melts relies on an experimentally measured shear viscosity that is free of the large errors introduced by undesirable effects, such as the temperature deviations due to viscous heating [3,6,7,8,9,10] or the pressure dependence of viscosity [11,12,13,14]. Capillary rheometry provides an efficient access to high-shear-rate flow properties relevant for processing. One of the major concerns in using a capillary rheometer to measure melt viscosity is viscous heating (or temperature thinning), although it can also occur at low shear rates in the presence of extremely viscous materials [5]. The standard methods for interpreting capillary rheometer data to determine the shear viscosity are, however, based on the isothermal flow condition. Because of the highly viscous nature of polymer melts and their low thermal conductivities, experimental and computational evidences for appreciable fluid temperature rises of 10–50 °C due to viscous heating have been reported in the literature for various materials in die flows [3,4,6,7,8,13,15,16,17,18,19,20,21], therefore viscous heating can be a disturbing factor in viscosity measurements.

To address viscous heating in capillary or slit flows, it is possible to measure the temperature rise of the fluid in the flow channels by means of various experimental techniques [4,15,16,17,18,19,21,22,23]. The effects of viscous heating can also be analyzed by means of theoretical calculations of temperature profiles [1,3,4,7,8,9,13,14,16,17,20,21,24,25,26,27,28,29,30,31,32]. In principle, the temperature rises obtained from either of the two approaches can be combined with a viscous heating correction method to retrieve the equivalent viscosity for isothermal flow [7,15,33]. Because of the coupling of the momentum and energy equations, the only way to obtain detailed temperature distributions in the general case is to numerically solve the governing equations along with the appropriate boundary conditions. To shed light on the effects of viscous heating on the experimentally measured shear viscosity, we not only performed 3-D nonisothermal capillary flow simulations to obtain information about the average fluid temperature rise, but also present a detailed viscous heating correction method that is supported by a theoretical derivation as well as by the nonisothermal flow simulation.

Some studies have investigated the influence of fluid temperature rise on measured shear viscosity, or have made a comparison between measured temperature rises and theoretical predictions. For example, using a calorimetric method, Daryanani et al., reported an equivalent shear viscosity corrected for the bulk temperature rises for a high density linear polyethylene [15]. They found that local temperature rises in the capillary can greatly exceed the bulk temperature rise (sometimes referred to as the cup-mixing temperature rise or the flow-average temperature rise). Cox and Macosko investigated viscous dissipation in various die flows for an acrylonitrile butadiene styrene (ABS) and a branched polyethylene [16]. They developed a mathematical model for the calculation of temperature profiles in the flow geometries using uncorrected shear viscosity. Reasonable agreement was obtained between the predicted and infrared measured melt surface temperature rises, and the average temperature rise estimated from the total mechanical energy input was shown to seriously underestimate the maximum temperature rise. An empirical method of correcting measurements of pressure in a capillary rheometer for viscous heating effects was developed by Kamal and Nyun for the case of adiabatic walls [13]. Their treatment may be considered as a generalized extension of the classical Rabinowitsch–Mooney method for estimating temperature-corrected viscosity in capillary flow. Friesenbichler and coworkers have recently carried out rheological measurements up to shear rates of $10^{6}{\;s}^{-1}$ for a polypropylene using a special rheological mold very similar to a standard injection mold [33]. By defining three thermal flow regimes according to the Cameron number (Ca), they also successfully calculated temperature corrected viscosity by taking into account a rise in average fluid temperature over the whole slit volume due to shear heating and compression heating [7,30,33]. In another work, they used a thermocouple to measure the temperature rise of a rubber compound issued from a conical die, and the measured temperatures were in good agreement with the prediction that takes into account both shear and elongational heating [17]. It should be apparent that most of the methods discussed above have contained simplifying assumptions. Furthermore, these methods have not been extensively tested against experimental capillary flow data, from which the uncorrected viscosity is determined. As a result, these methods have not met with complete success.

In this article we begin by demonstrating the necessity of performing the viscous heating correction in capillary rheometry for a series of thermoplastic melts that exhibit a distinct sensitivity of viscosity to temperature. On the basis of a newly derived expression for the average viscosity in nonisothermal flows, we elucidate the details of the correction of shear viscosity for fluid temperature rises. It appears to us, however, that no earlier studies have made a direct assessment of the generality of the viscous heating correction method proposed. For its simplicity and reliability, capillary rheometry provides a unique opportunity for extracting the viscous heating contribution to pressure drop by means of the 3-D nonisothermal flow simulation. It is shown that, in the shear-dominated capillary flow, the prediction of the viscous Cross model based on the temperature-corrected viscosity agrees favorably with experimental results. Furthermore, a near material-independent scaling relation is found and can be expressed in terms of the correlation between the dimensionless fluid temperature rise (Θ) and the Nahme number (Na), from which the temperature rise due to viscous heating as well as the resultant viscosity change can be predicted.

## 2. Materials and Methods

### 2.1. Thermoplastic Polymers and Rheological Characterization

We carried out a series of capillary flow experiments on five injection molding grade thermoplastic melts, which were chosen because of their very different sensitivity of viscosity to temperature at the high stress levels encountered in capillary rheometry (see their respective values of the temperature coefficient of viscosity $\alpha_{\eta }$ in Table 1). The polymer samples include a high impact grade polystyrene (HIPS, POLYREX PH-60, Chi Mei Corp., Tainan County, Taiwan), a general purpose polystyrene (GPPS, POLYREX PG-33, Chi Mei Corp.), an acrylonitrile butadiene styrene (ABS, POLYLAC PA-757, Chi Mei Corp.), a crystalline polypropylene homopolymer (PP, Globalene 6331, LCY Corp., Taipei, Taiwan), and a carbon-fiber reinforced polyamide 66 (PA66, LUVOCOM 1/XCF/25, LEHVOSS Group, Hamburg, Germany). Prior to the measurements, the pellets were dried under vacuum conditions overnight at temperatures suggested by the manufacturers. Steady shear flow measurements at low shear rates were performed using a rheometer (MCR 301, Anton Paar, Graz, Austria) equipped with a 25 mm diameter parallel-plate fixture (disposable aluminum substrate). A high pressure capillary rheometer (RG25, Göttfert, Buchen, Germany) was operated in the controlled speed mode to consecutively measure shear viscosities in the apparent shear rate range ${\dot{\gamma }}_{a}=50–5000$ $s^{-1}$. All the capillary measurements were repeated twice to ensure data reproducibility. Three circular dies of diameter D=1 mm and different lengths L=10, 20, and 30 mm were used to determine the entrance pressure drop (Bagley correction) and, hence, the true shear stress [34,35]. We found that the extrapolation method resulted in practically the same end corrections as those measured using a tapered orifice die (D=1 mm and L=0.2 mm). The Weissenberg–Rabinowitsch correction was performed to obtain the true wall shear rates [36,37]. Since the temperature dependences of the density ρ, specific heat capacity ${\hat{C}}_{p}$, and thermal conductivity k were taken into account in the nonisothermal flow simulation, the fluid properties ($\rho ,{\hat{C}}_{p},\;k$) were measured, respectively, using a PVT-6000 apparatus (Gotech), DSC 8500 (PerkinElmer), and RG25 accessory (Göttfert). Pressure–volume–temperature (PVT) diagrams for the molten materials were obtained under isobaric conditions (30, 60, 90, and 120 MPa) at a cooling rate of 5 °C/min. For the differential scanning calorimetry (DSC) measurements, each sample was heated to 250 or 300 °C at a temperature ramp rate of 10 °C/min in a nitrogen environment. The thermal conductivity measurements were performed under stationary conditions in a temperature range up to 250 or 300 °C. The measured values at an intermediate measurement temperature are compiled in Table 1 for reference, along with the melt flow index (MFI), the longest polymer relaxation time λ, and the temperature coefficient of viscosity $\alpha_{\eta }$ (=−∂η/η∂T) numerically evaluated at ${\dot{\gamma }}_{a}=500$ $s^{-1}$ from the temperature-corrected shear viscosity [1,25].

### 2.2. Governing Equations

The conservation equations for a compressible, generalized Newtonian fluid (GNF) in 3-D transient nonisothermal motion are [1]

(1) $$\frac{\partial \rho }{\partial t}+\nabla ∙\rho v=0$$

(2) $$\frac{\partial }{\partial t}\left(\rho v\right)+\nabla ∙\left(\rho vv+\tau \right)=-\nabla p+\rho g$$

(3) $$\rho {\hat{C}}_{p}\left(\frac{\partial T}{\partial t}+v∙\nabla T\right)=\nabla ∙\left(k\nabla T\right)+\eta {\dot{\gamma }}^{2}$$

where v is the velocity vector, τ is the total stress tensor, p is the pressure, g is the acceleration vector of gravity, η is the shear viscosity, and $\dot{\gamma }$ is the shear rate (the magnitude of the rate-of-strain tensor $\dot{\gamma }$). For a polymer melt modeled as a GNF, the stress tensor can be expressed as

(4) $$\tau =-\eta \left(\nabla v+\nabla v^{T}\right)$$

**Figure 1:**
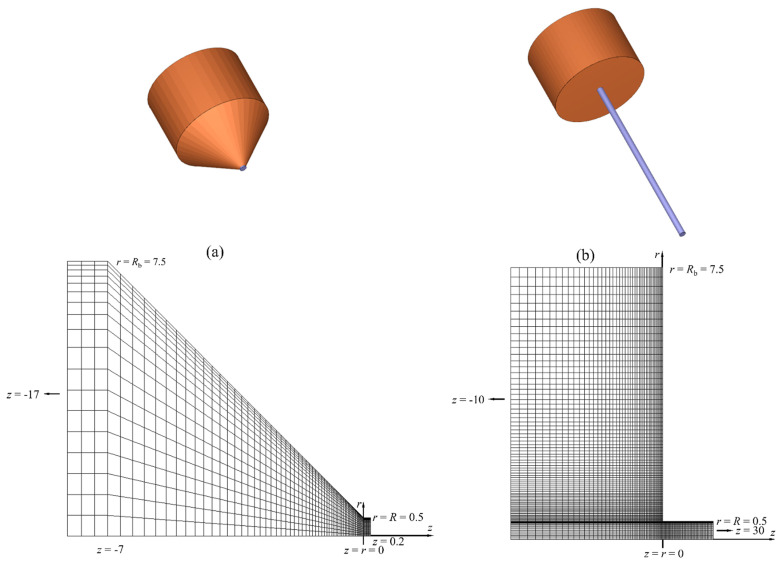
3-D and partial view of the two meshes used in the capillary flow simulations for (**a**) L/D=0.2 (D=1 mm and $2\phi =90^{{}^{\circ}}$) and (**b**) L/D=30 (D=1 mm and $2\phi =180^{{}^{\circ}})$.

The Cross viscosity model is employed to describe the polymer viscosity [38]

(5) $$\eta =\frac{\eta_{0}}{1+{\left(\frac{\eta_{0}\dot{\gamma }}{\tau^{*}}\right)}^{1-n}}$$

where $\eta_{0}$ is the zero-shear-rate viscosity, n is the power-law index, and $\tau^{*}$ is the critical stress upon which shear thinning commences. The temperature dependence of $\eta_{0}$ is expressed as the William–Landel–Ferry (WLF) equation, which has been found to hold for a wide variety of polymers [39,40]

(6) $$\eta_{0}=\eta_{0,r}\exp\left[\frac{-A_{1}\left(T-T_{r}\right)}{A_{2}+\left(T-T_{r}\right)}\right]$$

where $\eta_{0,r}$ is the zero-shear-rate viscosity at the reference temperature $T_{r}$, and $A_{1}$ and $A_{2}$ are the WLF coefficients. For simplicity, $T_{r}$ is taken to be the glass-transition temperature and $A_{2}=51.57$ is used [1]. The conservation equations of mass, momentum, and energy (Equations (1)–(3)) can be simultaneously solved, along with appropriate boundary conditions, to obtain the temperature, pressure, and velocity profiles for a compressible fluid under nonisothermal conditions by Moldex3D flow solver. We assumed fully developed flow at the inlet plane of the barrel and 1 atmosphere pressure at the outlet plane of the capillary exit (Figure 1). Two types of thermal boundary conditions were studied: (i) The isothermal wall boundary condition and (ii) the heat transfer boundary condition. The former assumes sufficiently effective cooling of isothermal die walls, while the latter accounts for the contact resistance between the die and the melt by applying Newton’s law of cooling with a heat transfer coefficient *h* (HTC).

### 2.3. Meshes for Capillary Flow Simulation

We performed the 3-D flow simulations in the capillary dies (Figure 1, L/D=0.2 and 30) with the viscous Cross-WLF model (Equations (5) and (6)). For the mesh arrangement, we chose a grid that progressively adds more elements toward the singularity in the entrance region of the capillary die, while the elements become bigger when going downstream of the inlet. A 3-D view and a partial view of the grids in the neighborhood of contraction are shown in Figure 1 for L/D=0.2 and 30. The domains represent a 15:1 circular contraction at an entry angle $2\phi =90\;\mathrm{or}\;180^{{}^{\circ}}$. Exponential biasing was used to generate elements in the axial and radial directions so as to reflect the large velocity gradients in the contraction regime and near the die wall. The grids were chosen after we confirmed that the subdivision of each element into four sub-elements to form denser grids gave virtually grid-independent results (less than 0.5% difference in the total pressure loss). Once the geometry was fixed, the only parameter left to vary was the apparent shear rate in the die ${\dot{\gamma }}_{a}=4Q/\pi R^{3}$, where Q is the volume flow rate and R is the die radius.

### 2.4. Temperature Profiles and Capillary Pressure Drops

**Figure 2:**
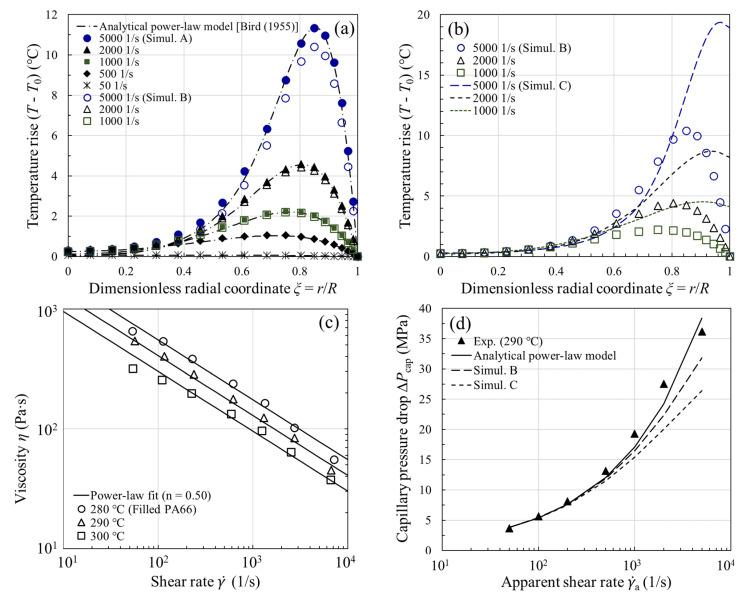
Calculated temperature profiles ($T-T_{0}$) under the (**a**) isothermal wall and (**b**) finite heat transfer boundary conditions for the filled PA66 melt at $T_{0}=290$ °C. The power-law fit to the uncorrected shear viscosity η ($\dot{\gamma }$) and the calculated capillary pressure drop $\Delta P_{\mathrm{cap}}$ are, respectively, shown in (**c**,**d**). Simulation A: η≠η(T) and isothermal wall; Simulation B: η=η(T) and isothermal wall; Simulation C: η=η(T) and finite heat transfer at the wall.

To validate the computed temperature profiles for capillary flow with viscous heating, we compared our simulation result with the analytical solution of a power-law fluid [1,3], assuming (i) the die wall is maintained at a fixed temperature $T_{0}$; (ii) the fluid is described adequately by a power-law viscosity, $\eta =m{\dot{\gamma }}^{n-1}$, with *m* and *n* independent of temperature; (iii) the velocity profile is fully developed at the die entrance; (iv) $\rho ,\;{\hat{C}}_{p},$ and k do not vary with temperature or pressure. An example of such calculation is provided for the filled PA66 in Figure 2a. The melt had the following physical properties at $T_{0}=290\;$°C (Table 1): $\rho {\hat{C}}_{p}=2.68\times 10^{6}\;J/m^{3}∙K$; k=0.282 W/m∙K; $m=4.05\times 10^{3}\;J/\mathrm{Pa}∙s^{n}$; n=0.50. The flow curve in Figure 2c manifests a power-law flow regime $\eta \sim {\dot{\gamma }}^{-\left(0.50\pm 0.05\right)}$. At a fixed axial distance z=15 mm (i.e., midpoint of the 30-mm-long capillary die depicted in Figure 1b), the temperature profiles at ${\dot{\gamma }}_{a}=50-5000$ $s^{-1}$ are plotted in Figure 2a. As our simulation (Simulation A) is subject to the same assumptions (i−iv) as in the analytical power-law model [1,3], the agreement between Simulation A (filled symbols) and the analytical solution (dash-dot lines) is remarkably good, except that the simulation predicts an extra temperature rise by ~0.5 °C near the tube center. The small discrepancy is caused by a small viscous heating contribution when the fluid undergoes a sudden contraction upon entering the tube (i.e., entrance effects). At high shear rates, there is a peak in the temperature profile near the wall where the velocity gradient and also the viscous heating are large. In this isothermal-wall case, where the heat transfer at the wall is extremely effective, the temperature rise can still be as large as ~12 °C. It is thus evident that viscous heating can produce nonignorable temperature rises in actual capillary rheometry.

In contrast to the analytical power-law model where the temperature-independent viscosity is assumed, we next took into account the temperature dependence of m in the same figure (Figure 2a, Simulation B). Since the temperature rise can now result in a lower viscosity (and hence less viscous heating), we find in Figure 2a that the temperature rises from Simulation B (open symbols) begin to fall below those from the analytical power-law model above a critical shear rate 1000 $s^{-1}$. The departure from the analytical power-law model indicates that the viscosity change, however, cannot be ignored if the temperature rise is more than a few degrees (~2 °C) [1]. Later, we demonstrate that the onset of viscous heating can be reliably predicted by the Nahme number [25,40,41], whose magnitude gives an indication of the relative importance of the rate of heat generation by viscous dissipation and the rate of heat removal by conduction at the capillary wall.

The temperature distribution in the example above is subject to the isothermal wall boundary condition. In fact, neither an isothermal nor an adiabatic wall exists [4,16]. In the next example, it was assumed that there is a finite amount of heat lost through the die wall. The heat transfer coefficient h at the polymer-mold interface is known to depend on a host of physical quantities (e.g., cavity pressure, surface roughness, etc.) [42], and the reported values in injection molding are in the range of 500−5000 $W/m^{2}∙K$ [43,44,45]. For simplicity, a constant heat transfer coefficient $h=1500\;W/m^{2}∙K$ was used throughout. When the temperature-dependent viscosity and the more realistic thermal boundary condition are simultaneously taken into account in the simulation (Simulation C, dashed lines), we see in Figure 2b that the corresponding temperature rise becomes more pronounced near the wall, where the dissipated energy is the maximum. Due to the contact resistance between the melt and the die, the temperature rise is more considerable than that of the isothermal wall (e.g., 20 °C vs. 10 °C at ${\dot{\gamma }}_{a}=5000{\;s}^{-1}$). In Simulation C, the maximum temperature rise at the tube exit (z=30 mm) can reach 24 °C, which explains why large surface temperature rises of extrudates were reported in the literature [16,19,23]. As the finite heat transfer boundary condition is physically more realistic, hereafter it is assumed in nonisothermal capillary flow simulations.

For capillary flow involving flow through a contraction of a certain entry angle, the total pressure drop $\Delta P_{\mathrm{total}}$ (from the barrel to the capillary exit) mainly consists of two components and may be written as [35,37]

(7) $$\Delta P_{\mathrm{total}}\approx \Delta P_{\mathrm{ent}}+\Delta P_{\mathrm{cap}}$$

where $\Delta P_{\mathrm{ent}}$ is the entrance pressure drop, which is mainly due to the elongational flow at the entrance, and $\Delta P_{\mathrm{cap}}$ is the pressure drop over the length L of the capillary, where the flow is shear-dominated; the pressure loss in the exit region of the capillary is presently ignored. Since the analytical power-law model is derived for a straight tube of radius R [1,3], for comparison with theory, we had to present our experimental and simulation results in terms of $\Delta P_{\mathrm{cap}}$ ($\approx \Delta P_{\mathrm{total}}-\Delta P_{\mathrm{ent}}$). In Figure 2d, the agreement between the measured $\Delta P_{\mathrm{cap}}$ and the analytical power-law model is simply fortuitous, whereas the discrepancy between experiment and the simulation (both Simulations B and C) is very likely due to the neglect of viscous heating correction of the shear viscosity for large temperature rises, in particular at high shear rates. In fact, the trend in the observed deviation in Figure 2d is universal for all the samples investigated in this study, and highlights the necessity of viscous heating correction to the shear viscosity.

## 3. Results and Discussion

### 3.1. Definition of Average Wall Shear Viscosity

**Figure 3:**
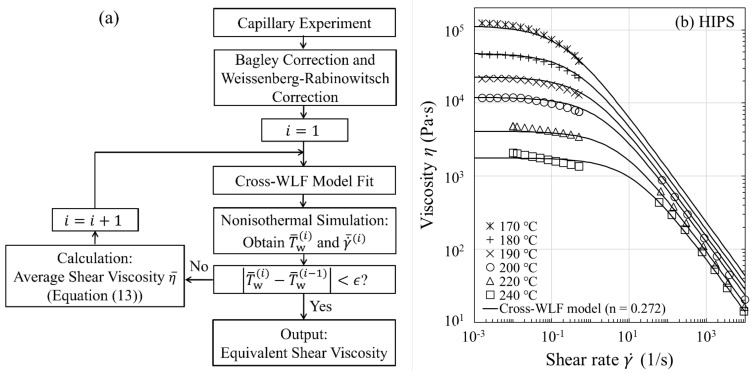
(**a**) Flow chart illustrating the viscous heating correction of the shear viscosity η ($\dot{\gamma }$). (**b**) Uncorrected steady-state shear viscosity η ($\dot{\gamma }$) for HIPS.

An average wall shear viscosity $\bar{\eta }$ that was used in the temperature correction method (Figure 3a) is defined here. For a straight capillary of uniform cross-section, we postulated a solution of the form $v_{z}=v_{z}\left(r\right)$, $v_{\theta }=0$, $v_{r}=0$, $P=P\left(z\right)$, and $T\left(r,\;z\right)$. The z-component of the equation of motion, in terms of τ, is [1,37]

(8) $$-\frac{dP}{dz}=\frac{1}{r}\frac{d}{dr}\left(r\tau_{rz}\right)$$

This may be integrated to give

(9) $$\tau_{rz}=-\frac{dP}{dz}\frac{r}{2}+\frac{C_{1}}{r}$$

The constant $C_{1}$ has to be zero, since one does not expect to have an infinite momentum flux at the axis of the tube. Here, dP is the pressure drop over a distance dz along the capillary. Equation (9) is therefore the result of the application of the equation of motion and is suitable for conditions where the fluid properties change appreciably along the capillary. At r=R, Equation (9) becomes

(10) $$\tau_{w}=-\frac{R}{2}{\frac{dP}{dz}|}_{r=R}$$

where $\tau_{w}$ is the wall shear stress. By integrating this equation over the capillary of length L and then applying the mean value theorem for definite integrals, we obtain the capillary pressure drop $\Delta P_{\mathrm{cap}}$ in nonisothermal flow

(11) $$\Delta P_{\mathrm{cap}}=\frac{2}{R}\int_{0}^{L}\tau_{w}\left(z\right)dz=\frac{2L}{R}\tau_{w}\left(z^{*}\right)=\frac{2L}{R}\tau_{w}\left(T^{*}\right)$$

where $z^{*}$ exists in [0,L] and $T^{*}$ is the local fluid temperature at $z^{*}$. We assume that $T^{*}$ is close enough to the mean fluid temperature ${\bar{T}}_{w}$, averaged over the wall surface along the tube, so that

(12) $$\Delta P_{\mathrm{cap}}\approx \frac{2L}{R}\tau_{w}\left({\bar{T}}_{w}\right)$$

Hereafter, the mean value along the die wall is designated by an overbar. Substitution of the material function $\tau_{w}\left({\bar{T}}_{w}\right)=\eta \left({\bar{T}}_{w}\right)\dot{\gamma }$ *(*${\bar{T}}_{w}$) into Equation (12) gives the following definition of the average shear viscosity $\bar{\eta }$ of fluids:

(13) $$\bar{\eta }=\eta \left({\bar{T}}_{w}\right)\approx \frac{R\Delta P_{\mathrm{cap}}}{2L\dot{\gamma }\left({\bar{T}}_{w}\right)}$$

We emphasize that the development here has been given for mean rheological properties of the fluids at the wall. As suggested by Equation (13), $\bar{\eta }$ may be better evaluated by using the mean fluid temperature at the wall ${\bar{T}}_{w}$, instead of the bulk temperature, as previously assumed. Throughout the iterative process described next in Figure 3a, Equation (13) was used to calculate $\bar{\eta }$, in which ${\bar{\tau }}_{w}$ ($=\tau_{w}\left({\bar{T}}_{w}\right)$) is set equal to the experimental wall shear stress and $\bar{\dot{\gamma }}$ ($=\dot{\gamma }\left({\bar{T}}_{w}\right)$) is determined from the simulation. It is worth mentioning that our simulation results presented later seem to lend support to the use of Equation (13) in nonisothermal capillary flows.

### 3.2. Temperature Correction of Shear Viscosity

**Figure 4:**
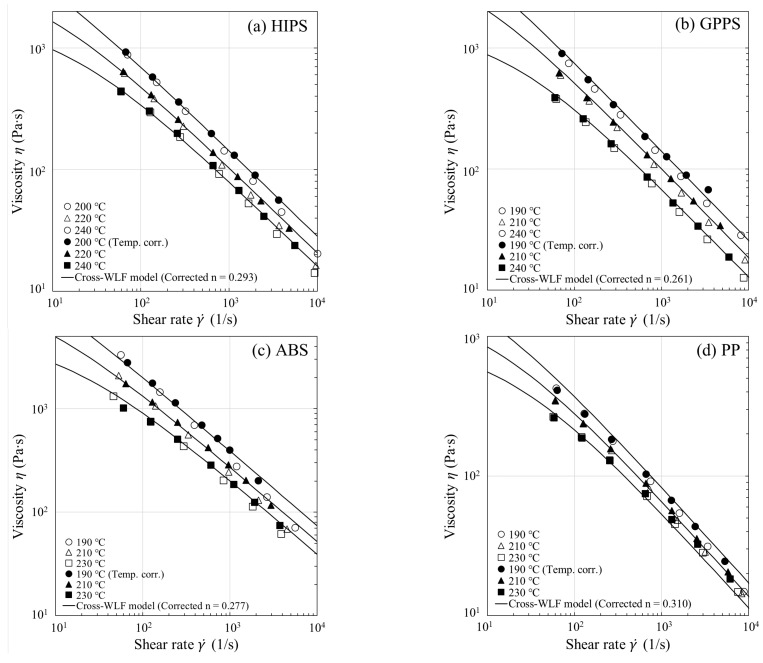

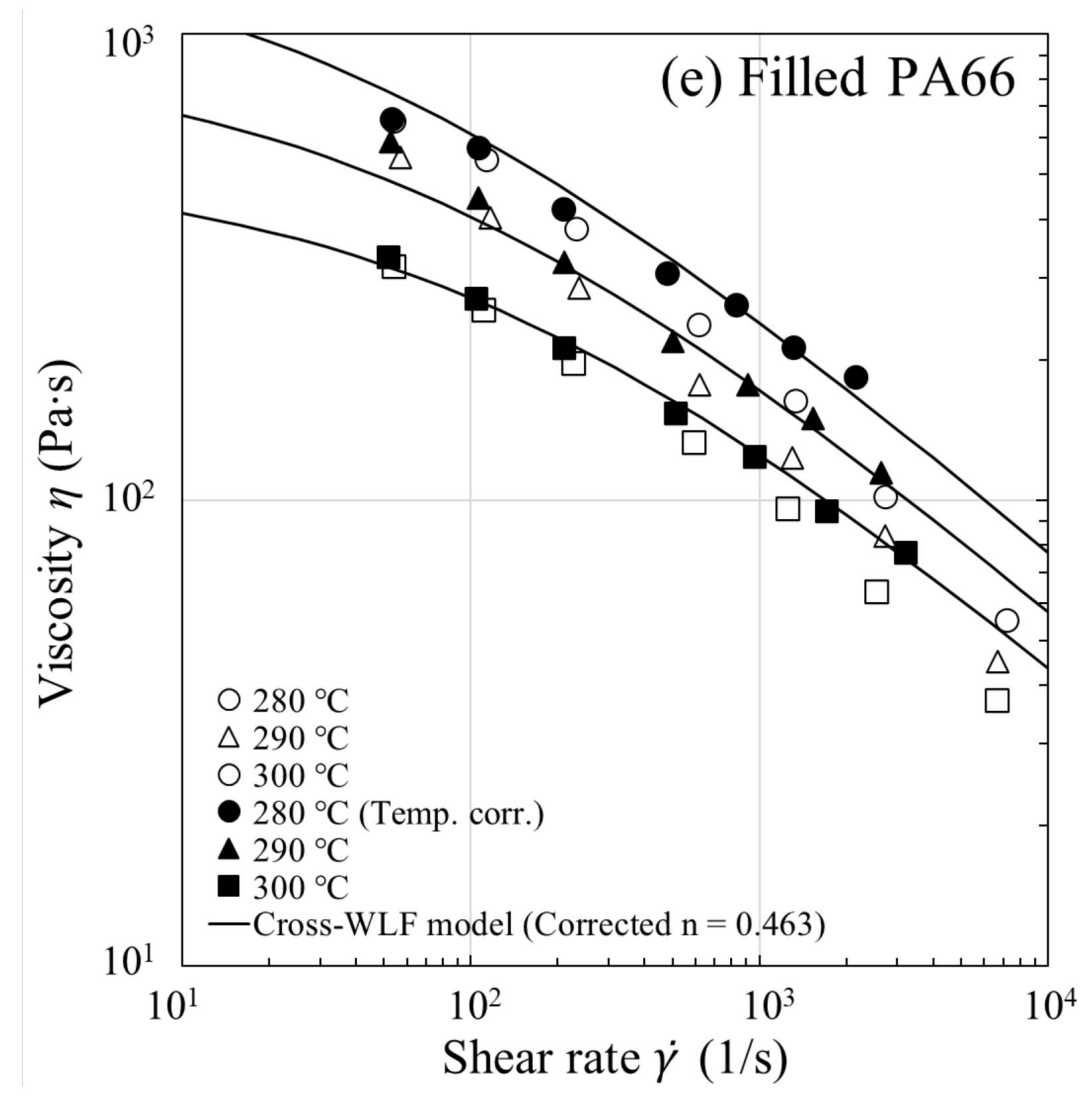
Steady-state shear viscosity η ($\dot{\gamma }$) with and without temperature correction for (**a**) HIPS, (**b**) GPPS, (**c**) ABS, (**d**) PP, and (**e**) filled PA66. The open and filled symbols represent, respectively, the uncorrected and the equivalent η ($\dot{\gamma }$). The Cross-WLF model fits based on the corrected *n* are shown by lines.

A flow chart detailing the approach for performing the correction of shear viscosity for fluid temperature rises is given in Figure 3a, where the Cross-WLF model is used to model the flow curve in both Newtonian and shear thinning regimes. As an illustration of the approach, we discuss the results for HIPS. The procedure for correcting the shear viscosity is as follows: (i) Apply, respectively, the Bagley correction and the Weissenberg-Rabinowitsch correction to the apparent viscosity data; (ii) Fit the uncorrected viscosity data determined from step (i) at the measurement temperatures ($T_{0}=170,\;180,\;190,\;200,\;220,\;\mathrm{and}\;240$ °C) to obtain an initial guess for the Cross-WLF model parameters (n, $\tau^{*}$, $\eta_{0,r}$, and $A_{1}$) in Equations (5) and (6) (see Figure 3b and the model parameters in Table 2); (iii) Perform the nonisothermal runs (ith iteration) at ${\dot{\gamma }}_{a}=50–5000$ $s^{-1}$ and $T_{0}=200,\;220,\;\mathrm{and}\;240$ °C to obtain the respective average fluid temperatures and shear rates at the capillary wall, and then replace the values from the (i−1)th iteration: with ${\bar{T}}_{w}^{\left(i-1\right)}$ replaced by ${\bar{T}}_{w}^{\left(i\right)}$ and ${\bar{\dot{\gamma }}}^{\left(i-1\right)}$ by ${\bar{\dot{\gamma }}}^{\left(i\right)}$ for each ${\dot{\gamma }}_{a}$; (iv) Calculate the average viscosities $\bar{\eta }$ using Equation (13) at fixed values of ${\bar{\tau }}_{w}$ (which are set equal to experimental values) and then fit $\bar{\eta }$ at the corresponding new fluid temperatures and shear rates to optimize the four Cross-WLF model parameters. Repeat steps (iii) and (iv) with improved model parameters until $\left|{\bar{T}}_{w}^{\left(i\right)}-{\bar{T}}_{w}^{\left(i-1\right)}\right|<\epsilon$ is satisfied, where the tolerance ϵ is set equal to 0.1 °C (usually 4−6 iterations suffice the purpose). Because the iterative process ensures self-consistency between the optimized Cross-WLF model parameters and the resultant temperature profiles, the approach ultimately yields a reliable equivalent shear viscosity (see Figure 4a and the corrected *n* value in Table 2). Considering that the viscosity curve is well into the power-law regime such as those shown in Figure 4a–e, one may need to correct the power-law index *n* only, due to the fact that the viscous heating occurring at high shear rates does not affect the onset of shear thinning ($\tau^{*}$), as well as the temperature dependence of the viscosity at low shear rates ($\eta_{0,r}$ and $A_{1}$).

For all the melts studied in Figure 4a–e, the equivalent shear viscosity at $T_{0}$ has been obtained by shifting the corrected shear viscosity, according to the WLF temperature shifting factor $a_{T}$, via the relations: $\eta \left(T_{0}\right)=\eta \left({\bar{T}}_{w}\right)/a_{T}$ and $\dot{\gamma }\left(T_{0}\right)=a_{T}\dot{\gamma }\left({\bar{T}}_{w}\right)$. The equivalent shear viscosity data are found to superpose onto the Cross-WLF model fit based on the corrected *n*. The temperature correction ultimately gives an equivalent viscosity that is more viscous (i.e., larger *n*) and will augment $\Delta P_{\mathrm{cap}}$. This can be seen from Equation (13) that $\bar{\eta }$ is proportional to $\Delta P_{\mathrm{cap}}$. Thus, as observed in Figure 4a–e, an enhancement of viscosity as large as ~30% at high shear rates could result in a similar increase in $\Delta P_{\mathrm{cap}}$, as discussed later in Figure 7.

### 3.3. Rheological Properties at Wall in Nonisothermal Flow

**Figure 5:**
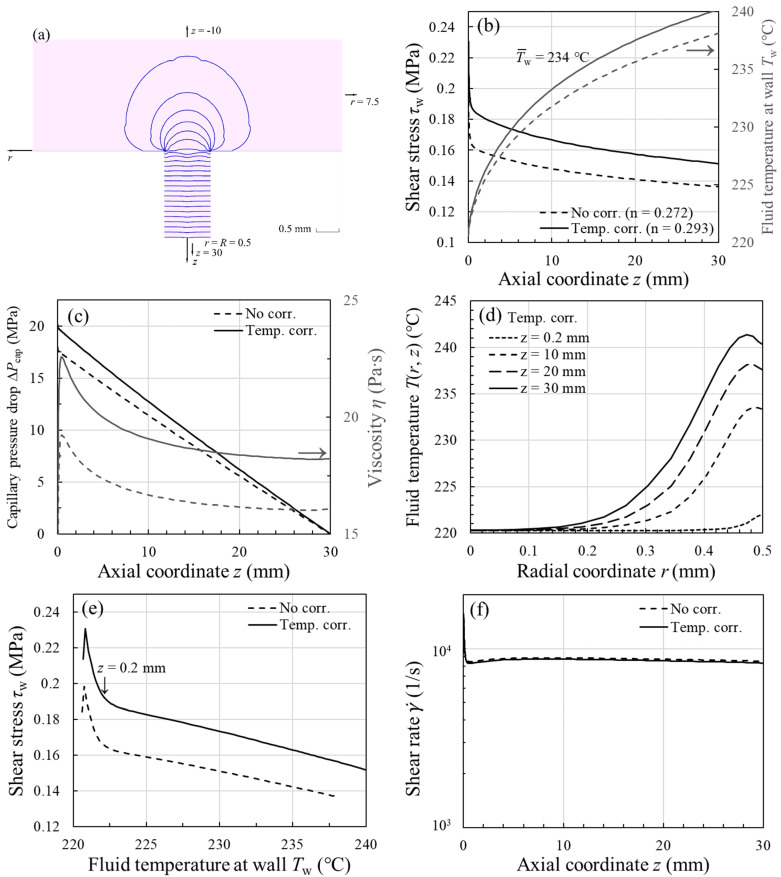
Graphs elucidating the simulated rheological properties along the capillary wall for HIPS at ${\dot{\gamma }}_{a}=5000\;s^{-1}$ and $T_{0}=220$ °C, based on η ($\dot{\gamma }$) with and without temperature correction: (**a**) Pressure iso-contours in the contraction region and axial variations of (**b**) shear stress $\tau_{w}$ and fluid temperature $T_{w}$, (**c**) capillary pressure drop $\Delta P_{\mathrm{cap}}$ and polymer viscosity η, (**d**) fluid temperature profile T(r, z), and (**f**) shear rate $\dot{\gamma }$. The data of (**b**) are replotted against $T_{w}$ in (e) to reveal an approximately linear relationship between $\tau_{w}$ and $T_{w}$.

Figure 5 shows the results of nonisothermal capillary flow simulations for HIPS, based on shear viscosities with and without temperature correction. Because, in both cases (*n* vs. corrected *n*), the trends in the rheological properties with axial coordinate *z* are very similar, we therefore focused on the simulated results based on the temperature-corrected shear viscosity (solid line) in this illustrative example. The simulation results for the other test polymers were qualitatively the same as those observed for HIPS and are hence not shown here. We can see in Figure 5a that, in the contraction region, the iso-contours of pressure become virtually independent of r after a small entrance length ~0.2 mm. This clear evidence is in support of the assumption made in the derivation of Equation (13) that only the axial variation in pressure is present (i.e., $P=P\left(z\right)$), whereas the radial variation is small enough to ignore (i.e., dP/dr≈0). As there is a sharp corner in the contraction regime, the steep stress gradient at z≈0 (see Figure 5b) is then associated with the singularity (i.e., high stresses generated at the entrance to the smaller channel). In Figure 5b, the shear stress $\tau_{w}$ and fluid temperature $T_{w}$ at the wall are plotted versus *z* for ${\dot{\gamma }}_{a}=5000{\;s}^{-1}$ and $T_{0}=220$ °C (z=0 corresponds to the die entrance), where it can be observed that $T_{w}$ increases in the flow direction due to viscous heating, while $\tau_{w}$ shows the opposite due to a progressively smaller pressure gradient (Figure 5c). The fluid temperature profiles $T\left(r,\;z\right)$ at different *z* are plotted in Figure 5d, where it can be seen that the fluid reaching one-third of the die length (i.e., z=10 mm) has attained a fluid temperature close to its average (${\bar{T}}_{w}=234$ °C), therefore $T_{w}$ does not vary in a linear fashion with *z*. Furthermore, the viscous heating causes a fluid temperature rise as large as ~20 °C at the tube exit (z=30 mm), clearly indicating the necessity of the temperature correction to the shear viscosity.

When $\tau_{w}$ is plotted versus $T_{w}$ in Figure 5e, we see a nearly linear relation between the two quantities, especially when the entrance length has been exceeded (i.e., z>0.2 mm). Thus, it explains why, in going from Equation (11) to (12), we have used the mean shear stress ${\bar{\tau }}_{w}$ and fluid temperature ${\bar{T}}_{w}$ in determining the mean shear viscosity $\bar{\eta }$. In Figure 5c, the change in viscosity after the correction is ~15%, therefore one can see a similar increment in the overall $\Delta P_{\mathrm{cap}}$ in the same figure, in accordance with Equation (13). With regard to the flow field, it can be seen from Figure 5f that the wall shear rate $\dot{\gamma }$ is not disturbed much by the temperature rise along the die, and that its average magnitude is close to that estimated by the Weissenberg–Rabinowitsch equation, which is 9600 $s^{-1}$. After applying the temperature correction to the shear viscosity, one can see in Figure 5e that the measured shear stress at ${\dot{\gamma }}_{a}=5000{\;s}^{-1}$ (${\bar{\tau }}_{w}=0.16\;\mathrm{MPa}$) can now correspond to the mean fluid temperature at the wall (${\bar{T}}_{w}=234$ °C), instead of the inlet temperature ($T_{0}=220$ °C). From the above analysis, we conclude that the simulation can provide the detailed rheological properties that would otherwise be difficult to access experimentally. Importantly, the simulation also lends support to the use of Equation (13) to determine $\bar{\eta }$ under nonisothermal conditions.

### 3.4. Assessment of Temperature-Corrected Shear Viscosity

**Figure 6:**
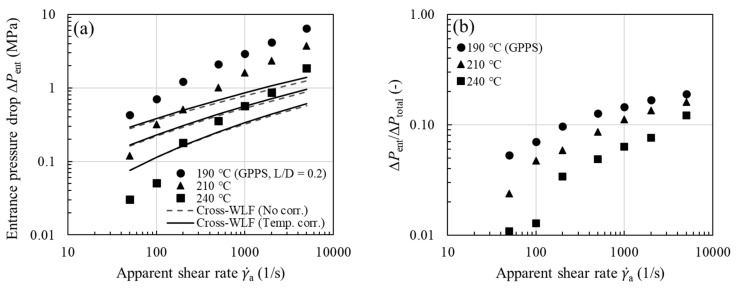
(**a**) Measured entrance pressure drop $\Delta P_{\mathrm{ent}}$ (symbols, L/D=0.2) vs. viscous Cross-WLF model prediction based on η ($\dot{\gamma }$) with and without temperature correction (lines) as a function of ${\dot{\gamma }}_{a}$ for GPPS. (**b**) Fraction of the entrance pressure drop $\Delta P_{\mathrm{ent}}$ for GPPS.

The effects of viscous heating (or temperature thinning) on experimentally measured shear viscosity are investigated for three types of thermoplastic melts, including amorphous polymers (HIPS, GPPS, and ABS), a crystalline polymer (PP), and a filled polymer (PA66). We began the nonisothermal capillary flow simulations with the viscous Cross-WLF model for the orifice die depicted in Figure 1a (L/D=0.2), based on the shear viscosities with and without temperature correction. It is known that the contraction flow has an elongational character along the centerline [35,37,46] and that a purely viscous model has a much lower elongational viscosity than a viscoelastic model, therefore it is not surprising to see that the viscous Cross-WLF model severely underpredicted the entrance pressure drop $\Delta P_{\mathrm{ent}}$ [35,47,48,49,50,51,52,53,54,55,56,57]. This is illustrated for GPPS in Figure 6a, where $\Delta P_{\mathrm{ent}}$ were measured using the 0.2-mm-long orifice die. As expected, $\Delta P_{\mathrm{ent}}$ increases significantly with increasing ${\dot{\gamma }}_{a}$, and its value is higher at lower measurement temperature. For the 30-mm-long die (depicted in Figure 1b), however, we see in Figure 6b that $\Delta P_{\mathrm{ent}}$ is not a small portion of the total; $\Delta P_{\mathrm{ent}}\;/\Delta P_{\mathrm{total}}$ can be as large as 0.1–0.2 at ${\dot{\gamma }}_{a}=5000{\;s}^{-1}$. Considering that viscous heating occurs mainly along the capillary die, and that the measured $\Delta P_{\mathrm{ent}}$ are available for all the test samples, in the following discussion, we concerned ourselves primarily with the pressure drop over the length of the capillary $\Delta P_{\mathrm{cap}}$ ($\approx \Delta P_{\mathrm{total}}-\Delta P_{\mathrm{ent}}$). The purpose of this analysis is two-fold: First, viscous heating contribution to $\Delta P_{\mathrm{cap}}$ can be better quantified without being influenced by the contraction flow. Second, the GNF is expected to hold well for the shear-dominated capillary flow.

**Figure 7:**
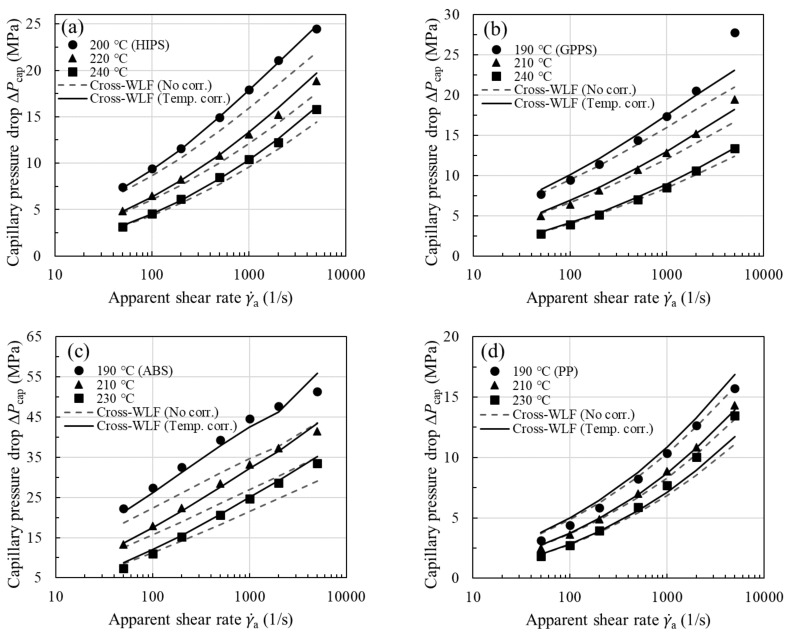

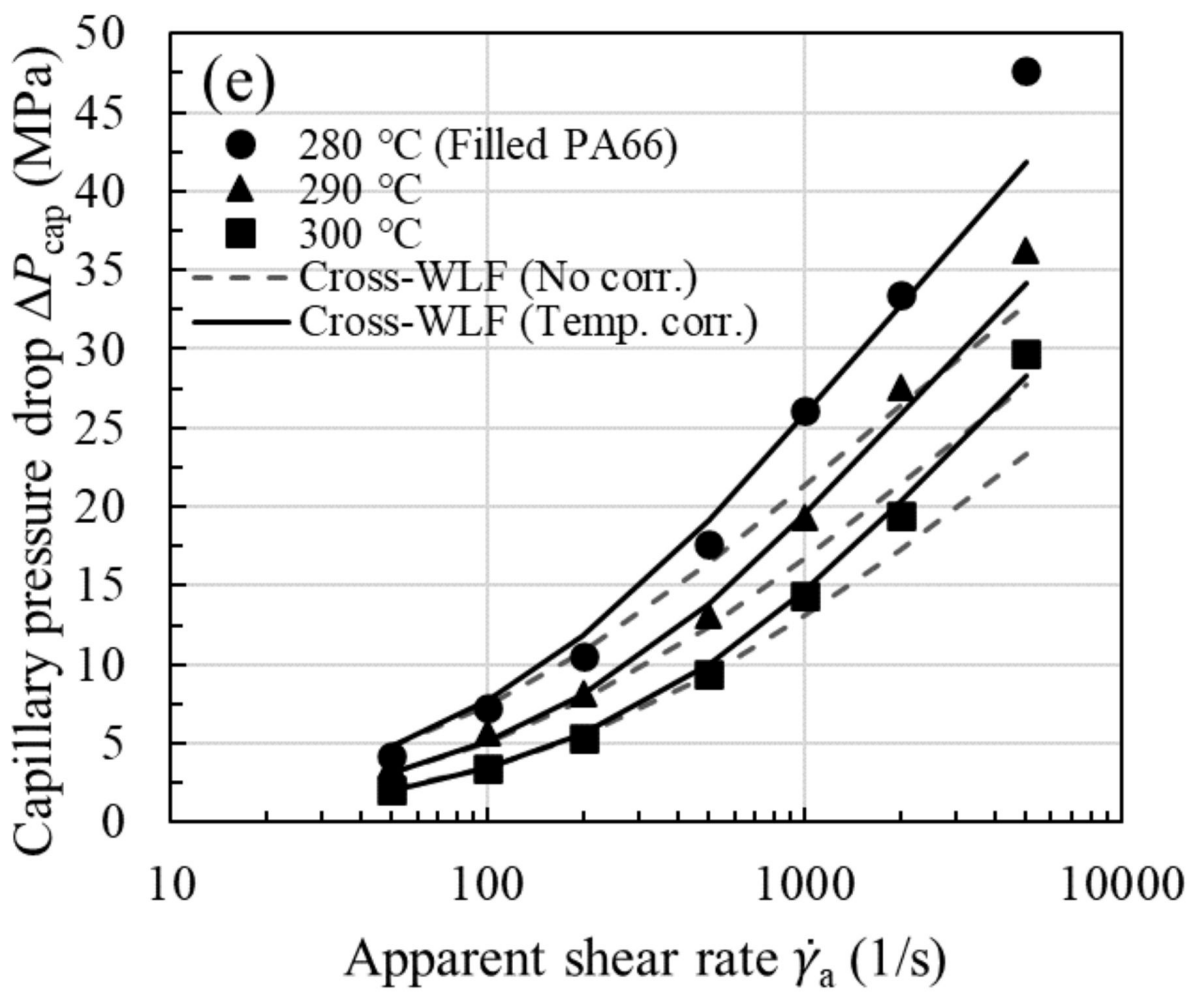
Measured capillary pressure drop $\Delta P_{\mathrm{cap}}$ (symbols, L/D=30) vs. viscous Cross-WLF model prediction based on η ($\dot{\gamma }$) with and without temperature correction (lines) as a function of ${\dot{\gamma }}_{a}$ for (**a**) HIPS, (**b**) GPPS, (**c**) ABS, (**d**) PP, and (**e**) filled PA66.

To evaluate the role of viscous heating in pressure loss, it is instructive now to consider the purely viscous Cross-WLF model and neglect viscous heating correction of shear viscosity (i.e., using the uncorrected viscosity). For all the samples studied, the experimental $\Delta P_{\mathrm{cap}}$ are compared with the Cross-WLF model predictions (dashed lines) in Figure 7a–e. Whereas the viscous model describes the measured pressure drops very well at low shear rates, it begins to underpredict the experimental data above a critical apparent shear rate (200 or 500 $s^{-1}$). As discussed later, the Nahme number (Na) can be used to delineate the flow regimes. In Figure 7a–e the trend in the observed deviation is universal for all the melts investigated and is reminiscent of the comparison made earlier in Figure 2d, where the likely underestimate of experimental $\Delta P_{\mathrm{cap}}$ is, to a large extent, ascribed to neglecting the effect of the fluid temperature rise on shear viscosity. Moreover, it can be seen in Figure 7c,e that, due to their large shear stresses and sensitivity of viscosity to temperature (i.e., large $\alpha_{\eta }$), the extent of disagreement is comparatively large for ABS and filled PA66.

**Figure 8:**
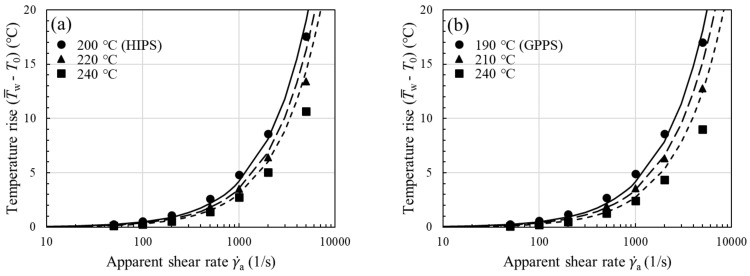

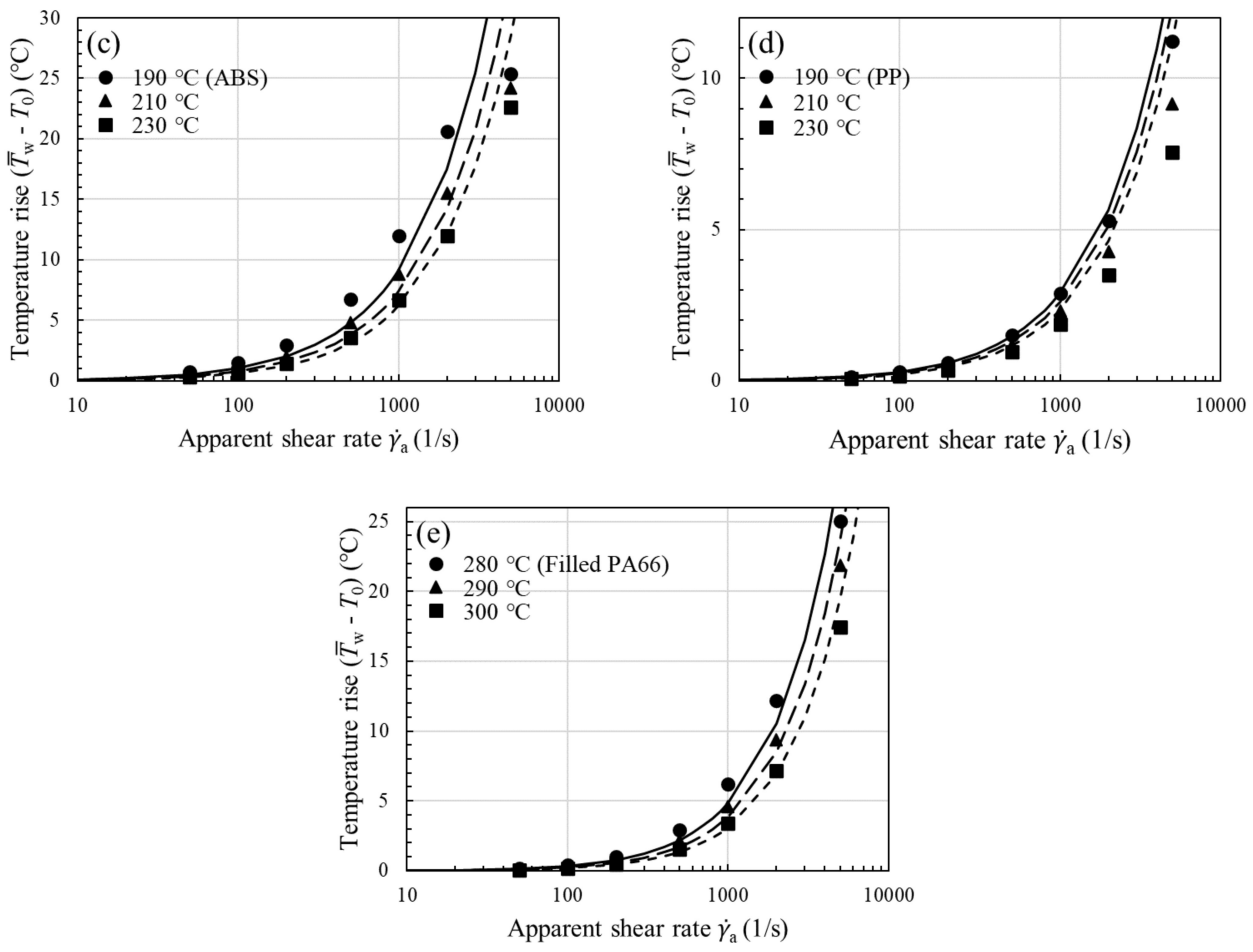
Temperature rise at the wall (${\bar{T}}_{w}-T_{0}$) vs. apparent shear rate ${\dot{\gamma }}_{a}$ for (**a**) HIPS, (**b**) GPPS, (**c**) ABS, (**d**) PP, and (**e**) filled PA66. Lines are predictions of Equation (18).

To show the viscous heating contribution to $\Delta P_{\mathrm{cap}}$, we next used the temperature-corrected shear viscosity in the nonisothermal capillary flow simulation; the corrected flow curves have been presented earlier in Figure 4 for all the samples studied. The increase in shear viscosity after performing the viscous heating correction is evident from the increased *n* value (see Table 2 for *n* vs. corrected *n*) and is reflected in larger $\Delta P_{\mathrm{cap}}$, in accordance with Equation (13). Using the equivalent viscosity $\bar{\eta }$ in the simulation, in Figure 7, we see a close agreement between experiment and model prediction (solid lines) over the entire range of shear rates. Thus, the proposed methodology in Figure 3a permits a reliable temperature correction for melts in capillary rheometry. Note once again that we have evaluated $\bar{\eta }$ in Equation (13) using the average fluid temperature at the wall ${\bar{T}}_{w}$ instead of the bulk fluid temperature, as previously assumed. Among the samples studied, viscous heating occurs to a significant degree for ABS and filled PA66, and the correction becomes clearly significant at the lowest measurement temperature. This can be easily understood to stem from the large fluid temperature rises at the wall as shown in Figure 8a–e (symbols), where it can be seen that viscous heating can result in pronounced deviations from the inlet temperature by 10–25 °C.

At this point, one might reasonably wonder whether or not the high-pressure effect on viscosity plays a minor role under the pressure conditions of these experiments. According to the Barus equation, $\eta_{0,p}=\eta_{0}\exp\left(\beta_{p}p\right)$ [58,59], the reciprocal of the pressure coefficient $\beta_{p}$ may indicate a critical pressure $p_{c}$, above which the pressure dependence of viscosity cannot be neglected. Reported $\beta_{p}$ values are $10<\beta_{p}\;\left({\mathrm{GPa}}^{-1}\right)<43$ for PS, $24<\beta_{p}\;\left({\;\mathrm{GPa}}^{-1}\right)<33$ for ABS, and $16<\beta_{p}\;\left({\mathrm{GPa}}^{-1}\right)<24$ for PP [33,60,61,62], corresponding to pressure ranges of $23<p_{c}\;\left(\mathrm{MPa}\right)<100$ for PS, $30<p_{c}\;\left(\mathrm{MPa}\right)<42$ for ABS, and $42<p_{c}\;\left(\mathrm{MPa}\right)<63$ for PP. For the materials studied in Figure 7, the measured $\Delta P_{\mathrm{cap}}$ are comparatively small, so that the pressure dependence of viscosity may be safely neglected in the present analysis.

### 3.5. Material-Independent Scaling Relation

In capillary rheometry, it is of practical importance to know the onset of nonisothermal flow. A dimensionless group was used for characterizing the severity of viscous heating in the individual melts. In fact, the equation of motion (Equation (2)) and the equation of energy (Equation (3)) are coupled through the temperature-dependent viscosity. The extent of the coupling increases with the value of the Nahme number [25,40,41,63]

(14) $$\mathrm{Na}={\bar{v}}_{z}^{2}\alpha_{\eta }\eta^{0}/k$$

where ${\bar{v}}_{z}$ is the average axial velocity ($=4Q/\pi R^{2}$), $\alpha_{\eta }$ is the temperature coefficient of viscosity (=−∂η/η∂T), and $\eta^{0}$ is the characteristic viscosity evaluated at a characteristic shear rate (${\bar{v}}_{z}/R$) and characteristic temperature ($T^{0}$). Equation (14) can be rewritten in terms of the familiar ${\dot{\gamma }}_{a}$ as $\mathrm{Na}=R^{2}{\dot{\gamma }}_{a}^{2}\alpha_{\eta }\eta^{0}/k$. It can be seen from the relation that, at a given ${\dot{\gamma }}_{a}$, $\mathrm{Na}\sim R^{2}$, whereas, at a given Q, $\mathrm{Na}\sim R^{-4}$. The former case has been demonstrated for flow of a polymer melt through two capillaries of different diameters but the same L/R [41], while the later indicates that the viscous heating occurs to a significant degree when R is very small.

**Figure 9:**
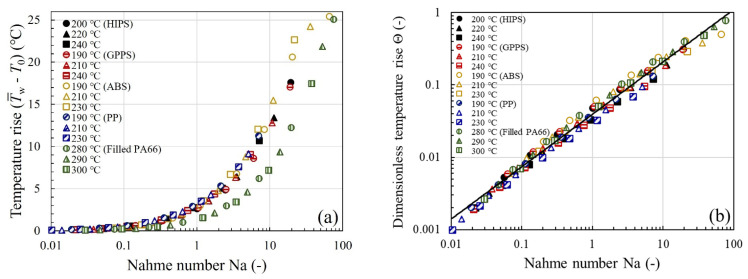
(**a**) Temperature rise $\left({\bar{T}}_{w}-T_{0}\right)$ and (**b**) dimensionless temperature rise $\Theta =\alpha_{\eta }\left({\bar{T}}_{w}-T_{0}\right)$ vs. Nahme number Na for the five thermoplastic melts studied. Best-fit line through the data supports the approximate scaling relation $\Theta \sim {\mathrm{Na}}^{0.72}$.

For a self-consistent analysis of the calculated temperature rises in Figure 8 and Figure 9, the Na number has been determined using $\alpha_{\eta }$ and $\eta^{0}$ that are numerically evaluated from the temperature-corrected viscosity, not the uncorrected one. The Na number compares the viscous dissipation term and the conduction term in Equation (3). For values of Na greater than 0.5–1 (depending on the particular die geometry and thermal boundary conditions), the viscous dissipation can cause nonignorable viscosity changes. Indeed, when the temperature rises of Figure 8 are replotted in terms of the Na number, as in Figure 9a, they begin to increase rapidly upon the onset of viscous heating at Na≈1. Thus, the transition is correctly captured by our simulation. Among the materials studied, ABS and filled PA66 can attain a comparatively large Na number ~70 at the highest shear rate, therefore the viscosity augmentation (hence increased pressure loss), after the viscous heating correction, is the most significant (Figure 4c,e or Figure 7c,e). By contrast, due to the small Na number ~7 it attains, PP requires a small correction to the viscosity.

Since the Nahme number is a ratio of the temperature rise characteristic of the viscous heating in the capillary flow problem to the temperature change necessary to alter the viscosity [1,41], we next introduced a quantity, $\Theta =\alpha_{\eta }\left({\bar{T}}_{w}-T_{0}\right)$, made dimensionless with $\alpha_{\eta }$, and the data of Figure 9a are replotted in Figure 9b. Interestingly, we find that the effect of viscous heating on temperature rise can be summarized in Figure 9b by a near material-independent scaling relation

(15) $$\Theta =c_{1}{\mathrm{Na}}^{c_{2}}$$

where $c_{1}=0.039$ and $c_{2}=0.72$. This finding may imply a strong correlation between the dimensionless temperature rise and the Na number for all the thermoplastic melts investigated in this study. In obtaining Equation (14), it has been assumed that, over small ranges of temperature, the viscosity varies with temperature as [25,40,64]

(16) $$\eta =\eta^{0}\exp\left[-\alpha_{\eta }\left(T-T_{0}\right)\right]$$

Thus, a rise in fluid temperature can result in a reduced fluid viscosity by the relative amount

(17) $$\frac{\eta^{0}-\eta }{\eta^{0}}=\Theta =\alpha_{\eta }\left({\bar{T}}_{w}-T_{0}\right)$$

Therefore, Θ may be interpreted as the ratio of the amount of viscosity correction ($\eta^{0}-\eta$) to the uncorrected viscosity $\eta^{0}$. Accordingly, in Equation (15), the power-law dependence manifested by Θ can be interpreted as follows: An increase in the Na number causes the fluid temperature rise due to viscous heating as well as the resultant viscosity change (or viscosity correction needed). For instance, when Na=1 and 10, the maximum percentage errors in the viscosity are approximately 5 and 20%, respectively.

To check the prediction of the power-law scaling relation (Equation (15)) for the temperature rises reported in Figure 8, we substitute the expression for the Na number into Equation (15) and get

(18) $${\bar{T}}_{w}-T_{0}=\frac{c_{1}}{\alpha_{\eta }}{\left(\frac{R^{2}{\dot{\gamma }}_{a}^{2}\alpha_{\eta }\eta^{0}}{k}\right)}^{c_{2}}$$

The derived correlation can be directly applied to correct the melt viscosities from a capillary die having similar dimensions so as to minimize the computational effort required. Here, an expression for $\alpha_{\eta }$ in the power-law regime can be found by equating the temperature shifting factors in Equations (6) and (16), and one arrives at the following,

(19) $$\alpha_{\eta }=\frac{nA_{1}\left(T-T_{r}\right)}{\left(A_{2}+T-T_{r}\right)\left(T-T_{0}\right)}$$

in which the values of the Cross-WLF model parameters can be used. For an accurate determination of $\alpha_{\eta }$, $T_{r}$ needs to be close to $T_{0}$. For n=1, Equation (19) reduces to the expression for Newtonian fluids. Equation (18) is for the convenience of making calculations and to show the extent of agreement with the simulation data. The temperature increase determined from the equation can be seen in Figure 8 (lines), where the accuracy of the predictions is acceptable for most of the materials studied. For some purposes, the prediction of Equation (18) for the mean fluid temperature rise in thermally developing capillary flows may be adequate, especially for order-of-magnitude estimates.

## 4. Conclusions

We have systematically investigated the viscous heating correction (or the temperature thinning effect) and extracted its contribution to the capillary pressure drop in thermally developing capillary flows for a series of injection molding grade thermoplastic melts (amorphous, crystalline, and filled polymers). To highlight the importance of applying the temperature correction to the shear viscosity, we begin by showing that the power-law model based on the uncorrected viscosity substantially underestimates the capillary pressure drop $\Delta P_{\mathrm{cap}}$ (Figure 2d), and that the trend in the observed deviation is universal for all the melts investigated (Figure 7a–e). A viscous heating correction method for determining the equivalent viscosity is then developed to take into account the considerable temperature rises. As suggested by the theoretical derivation and the 3-D nonisothermal simulations, the average shear viscosity $\bar{\eta }$ has to be evaluated by using the average fluid temperature at the wall ${\bar{T}}_{w},$ rather than the bulk temperature, as previously assumed. Only by performing the simulations is it possible to obtain the detailed temperature profiles over the capillary die, which would be otherwise difficult to access experimentally.

The subtraction of the entrance pressure drop $\Delta P_{\mathrm{ent}}$ from the total pressure drop $\Delta P_{\mathrm{total}}$ allows us to investigate the viscous heating contribution to the capillary pressure drop $\Delta P_{\mathrm{cap}}$ in the shear-dominated capillary flow. To assess the generality of the temperature correction, we applied the approach to several polymer melts, and the predictions of the viscous Cross-WLF model are found to be in good to excellent agreement with experimental $\Delta P_{\mathrm{cap}}$. In every case, the onset of viscous heating is observed to coincide with Na≈1 and corresponds to a temperature rise of a few degrees (~2 °C). We also report a strong correlation between the dimensionless fluid temperature rise and the Nahme number that can be summarized by a near material-independent scaling relationship: $\Theta \sim {\mathrm{Na}}^{0.72}$, from which the fluid temperature rise due to viscous heating as well as the resultant viscosity change can be predicted. The inferred correlation may not only be directly applied to correct the melt viscosities from a capillary die having similar dimensions, but also lend additional support to the idea that $\bar{\eta }$ has to be evaluated by using ${\bar{T}}_{w}$.

Judging from its success in retrieving the reliable equivalent shear viscosity from the 3-D nonisothermal capillary flow simulation, the methodology described in this study may be applicable to dies of different lengths or radii, or of an annular or slit cross-section, where the viscous heating becomes an issue. As the calculated fluid temperature rise at the wall highly depends on the thermal boundary conditions specified, it would be important to combine experimental and numerical approaches to determine the heat transfer coefficient at the polymer-mold interface, as well as to study its influence on the capillary flow data.

## Figures and Tables

**Table 1:** Fluid properties of polymer melts.

Sample	$T_{0}$ (°C)	$\rho {\hat{C}}_{p}$ $\;(J/m^{3}∙K)$	*k* (W/m∙K)	$\mathrm{MFI}\;(g/10\;\min){\;}^{†}$	$\lambda \;\left(s\right)$	$\alpha_{\eta }$ (1/K)
HIPS	220	$2.17\times 10^{6}$	0.256	5.5	0.178	$1.51\times 10^{-2}$
GPPS	210	$2.08\times 10^{6}$	0.259	8.0	0.250	$1.53\times 10^{-2}$
ABS	210	$2.27\times 10^{6}$	0.235	1.6	0.222	$1.68\times 10^{-2}$
PP	210	$2.05\times 10^{6}$	0.279	14.5	0.109	$1.14\times 10^{-2}$
Filled PA66	290	$2.68\times 10^{6}$	0.282	20.8	0.015	$3.44\times 10^{-2}$

^†^ HIPS, GPPS, and ABS: 200 °C/5.0 kg; PP: 230 °C/2.16 kg; filled PA66: 290 °C/5.0 kg.

**Table 2:** Cross-WLF model parameters for polymer melts.

Sample	*n*	Corrected *n*	$\tau^{*}\;(\mathrm{Pa})$	$\eta_{0,r}$ (Pa∙s)	$T_{r}\;(K)$	$A_{1}$	$A_{2}$
HIPS	0.272	0.293	$2.30\times 10^{4}$	$5.94\times 10^{11}$	373.15	26.86	51.57
GPPS	0.246	0.261	$2.42\times 10^{4}$	$1.94\times 10^{13}$	363.15	31.29	51.57
ABS	0.237	0.277	$6.17\times 10^{4}$	$7.14\times 10^{13}$	363.15	31.99	51.57
PP	0.299	0.310	$1.59\times 10^{4}$	$3.92\times 10^{19}$	263.15	46.48	51.57
Filled PA66	0.368	0.463	$6.02\times 10^{4}$	$1.73\times 10^{25}$	373.15	65.22	51.57

## Data Availability

The data presented in this study are available on request from the corresponding author.
